# Measures of Physical Activity Using Cell Phones: Validation Using Criterion Methods

**DOI:** 10.2196/jmir.1298

**Published:** 2010-01-29

**Authors:** Christin Bexelius, Marie Löf, Sven Sandin, Ylva Trolle Lagerros, Elisabet Forsum, Jan-Eric Litton

**Affiliations:** ^3^Clinical Epidemiology UnitDepartment of MedicineKarolinska InstitutetStockholmSweden; ^2^Department of Clinical and Experimental MedicineFaculty of Health ScienceLinköping UniversityLinköpingSweden; ^1^Department of Medical Epidemiology and BiostatisticsKarolinska InstitutetStockholmSweden

**Keywords:** Physical activity, cellular phone, epidemiological methods, data collection

## Abstract

**Background:**

Physical activity is associated with reduced risks of many chronic diseases. Data collected on physical activity in large epidemiological studies is often based on paper questionnaires. The validity of these questionnaires is debated, and more effective methods are needed.

**Objective:**

This study evaluates repeated measures of physical activity level (PAL) and the feasibility of using a Java-based questionnaire downloaded onto cell phones for collection of such data. The data obtained were compared with reference estimates based on the doubly labeled water method and indirect calorimetry (PAL_ref_).

**Method:**

Using a Java-based cell phone application, 22 women reported their physical activity based on two short questions answered daily over a 14-day period (PAL_cell_). Results were compared with reference data obtained from the doubly labeled water method and indirect calorimetry (PAL_ref_). Results were also compared against physical activity levels assessed by two regular paper questionnaires completed by women at the end of the 14-day period (PAL_quest1_ and PAL_quest2_). PAL_cell_, PAL_quest1_, and PAL_quest2_ were compared with PAL_ref_ using the Bland and Altman procedure.

**Results:**

The mean difference between PAL_cell_ and PAL_ref_ was small (0.014) with narrow limits of agreement (2SD = 0.30). Compared with PAL_ref_, the mean difference was also small for PAL_quest1_ and PAL_quest2_ (0.004 and 0.07, respectively); however, the limits of agreement were wider (PAL_quest1_, 2SD = 0.50 and PAL_quest2_, 2SD = 0.90). The test for trend was statistically significant for PAL_quest1_ (slope of regression line = 0.79, *P* = .04) as well as for PAL_quest2_ (slope of regression line = 1.58, *P* < .001) when compared with PAL_ref_.

**Conclusion:**

A Java-based physical activity questionnaire administered daily using cell phones produced PAL estimates that agreed well with PAL reference values. Furthermore, the limits of agreement between PAL obtained using cell phones, and reference values were narrower than for corresponding estimates obtained using paper questionnaires. Java-based questionnaires downloaded onto cell phones may be a feasible and cost-effective method of data collection for large-scale prospective studies of physical activity.

## Introduction

Physical activity is associated with reduced risks of several chronic diseases such as cardiovascular diseases, diabetes, and certain cancers [1,2]. To further explore the preventive effects of physical activity, more epidemiological studies with solid data collection are needed. Because energy expenditure affects energy balance and thus body weight and composition, an important health-related consequence of physical activity is the amount of energy expended when being physically active. Energy expenditure in response to physical activity can be measured as total energy expenditure divided by basal metabolic rate (physical activity level, PAL) or as total energy expenditure minus basal metabolic rate (activity energy expenditure, AEE).

In free-living subjects the best method of assessing PAL and AEE is to combine measurements of total energy expenditure assessed using the doubly labeled water method with measurements of basal metabolic rate assessed using indirect calorimetry [3-5]. In the doubly labeled water technique [6], carbon dioxide production is estimated as the difference between the turnover rates of two tracer isotopes (^2^H and ^18^O) in the body water pool from which energy expenditure is calculated[6]. The method has been successfully validated in human subjects [6] and is unique since it can assess energy expenditure over a period of days and weeks in free-living individuals with minimal interference with daily life activities. However, this method of assessing PAL and AEE using the combination of the doubly labeled water method and indirect calorimetry was developed for clinical settings and is less suitable for larger population-based studies [4].

Other available methods of assessing energy expenditure due to physical activity are activity records, heart rate monitors, and accelerometers [4,5]. The accuracy of these methods is debated, and they are demanding for study participants and study personnel. The method most commonly used by epidemiologists is to administer paper questionnaires where participants report their physical activity retrospectively. Such questionnaires are easy to use and non-invasive [7-13] but are prone to bias since they rely on memory [14]. Neilson et al [15] reviewed a large number of studies where AEE estimates obtained using questionnaires had been compared with reference estimates based on the doubly labeled water method and indirect calorimetry. They concluded that most questionnaires are not sufficiently valid to assess AEE, indicating the need for new approaches within this area [15]. Telecommunication technologies have created possibilities for accurate real-time data collection and may thus reduce information bias due to retrospective data collection [16]. The high access to cell phones makes these devices potential tools for large-scale data collection in population-based studies, and previous studies have demonstrated the feasibility of collecting data using short message service (SMS), or text, messaging through cell phones [17,18]. SMS has been used in intervention trials, therapy management systems [19,20], as well as studies involving physical activity and weight loss [21-26]. With cell phones connected to the Internet, Web-like applications and more advanced questionnaires resembling Web-based questionnaires are also made possible [27-29].

The primary aim of this study was to validate PAL values aggregated over 14 days against reference estimates based on the doubly labeled water method and indirect calorimetry. The aim was also to evaluate the feasibility of collecting repeated measures of PAL through a Java-based questionnaire downloaded onto cell phones. Two traditional paper questionnaires were also used to compare the validity of the PAL estimates based on questionnaires administered daily over cell phones with the validity of the PAL estimates based on paper questionnaires administered retrospectively.

## Methods

### Subjects and Study Overview

Twenty-two healthy women were recruited to a study on energy metabolism and physical activity by means of advertisements in the local press between September 2007 and February 2008. The women were employed in different areas of work, such as office work, nursing, and childcare. On the morning of the first day, basal metabolic rate was measured. Each woman was then given a dose of stable isotopes and asked to collect urine samples during the subsequent 14-day period to measure total energy expenditure. (Measurement of metabolic rate, isotope administration, urine collection, and urine analysis are explained in more detail below.) A reference PAL (PAL_ref _) covering this 14-day period was calculated as total energy expenditure divided by basal metabolic rate.

Each woman was instructed over the phone in how to download the Java-based questionnaire. Before the installation, the women were registered on a study-specific website. If a woman’s cell phone didn’t support the Java script, the study center lent her a phone that did. During the 14-day period, each woman received a text message every day at 9pm to remind her to answer the two questions about her physical activity during the same day through the Java-based questionnaire on her cell phone. The two questions were: (1) How physically active have you been during work/daytime today? and (2) How physically active have you been during leisure time/evening today? (Table 1). Answers to the questions were delivered to the study center in real-time through the cell phone’s online connection and could be monitored on the study-specific website. If a woman did not reply, the study center sent her an additional SMS as a reminder. At the end of the 14-day period, each woman delivered the urine samples to the study center and while there answered two paper questionnaires regarding her physical activity during the preceding 14-day period. The study was designed according to the Helsinki Declaration and was approved by the Central Ethical Review Board in Stockholm, Sweden.

**Table 1:** The two questions administered using participants’ cell phones

	Answer Category	PAL Score
**1. How physically active have you been during work/daytime today? **		PAL: Derived Score for each Category[30]^a ^
	a. Mostly sitting	1.55
	b. Sitting/standing/walking	1.65
	c. Standing/walking most of the time	1.85
	d. Heavy work	2.2
**2. How physically active have you been during leisure time/evening today? **		Additional Contribution to PAL [31]^a ^
	a.Mostly sitting	+0
	b. Light/walking 30min	+0.06
	c. Moderate/cycling≥30min	+0.15
	d. Sport/cycling≥60min	+0.29

^a^See text for an explanation of how PAL was calculated from cell phone questions

### Cell Phone Application

**Figure 1:**
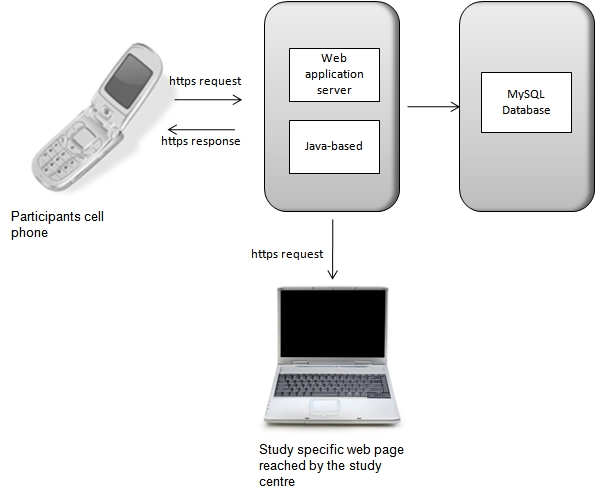
Schematic figure of the data communication between the cell phone, web application server, study-specific webpage, and the database

The download of the Java-based cell phone application was initialized through a WAP-push message. The Web client is built on Ajax in the Wicket application. The Web and application server was developed on Spring to build and run Java application and Hibernate to perform object relation mapping and query to the database. The database for collected information was MySQL, and Linux was used as the operating system. All data traffic was through https (hypertext transfer protocol secure). The procedure is described in Figure 1.

### Measurement of basal metabolic rate and total energy expenditure

Basal metabolic rate was measured in the following manner. On the morning of the first day of the study, each woman’s carbon dioxide production and oxygen consumption were measured after an overnight fast and 45 minutes of rest by indirect calorimetry during a 20-minute period using a ventilated hood system (Deltratrac Metabolic Monitor, Datex Instrumentarium Corp, Helsinki, Finland). The women came to the hospital by car to keep physical activity to a minimum. Carbon dioxide production and oxygen consumption were converted to basal metabolic rate through the equation by Weir [32].

To obtain measures of total energy expenditure, each woman was given an accurately weighed dose of the stable isotopes deuterium and oxygen-18 (0.09 g^2^H_2_0 per kg body weight and 0.23 g H_2_^18^0 per kg body weight) after having collected two to three background urine samples. Women collected another six urine samples on days 1, 4, 6, 8, 11, and 15 after the day of dosing. Isotopic enrichments of dose and urine samples were analyzed as described by Lof et al [33]. Analytical precision for results expressed in ppm was 0.22 for^2^H and 0.03 for^18^O. Carbon dioxide production was calculated as described by Coward [34] with deuterium and oxygen-18 dilution spacesas the distribution spaces matching their respective rate-constants for isotopic disappearance and assuming 30% of water losses to be fractionated. The ratio between deuterium-space and oxygen-18 space was 1.033 ± 0.006. Total metabolic rate was calculated from carbon dioxide production assuming the food quotient to be 0.85 [35]. For one subject, the same dose and urine samples were analyzed nine times. The following coefficients of variation were obtained: 1.2% for total energy expenditure, 0.3% for total body water, and 0.15% for the ratio between deuterium-space and oxygen-18 space.

### Calculation of PAL From Cell Phones

Women’s answers to the two short questions about physical activity during the same day were converted to PAL according to the values shown in Table 1 that corresponded to participants’ responses. All data were displayed on the study-specific website and stored in the local database. The two questions were derived in order to cover work/daytime and leisure time/evening activities. To our knowledge, these specific questions have not been used before, but a similar approach has been used in other studies [36,37]. PAL was calculated daily by adding the PAL value obtained from question 1 to the contribution of PAL from question 2. The PAL values for each option during work/day time (question 1) were obtained from Black et al [30], and the contribution to PAL from activities during leisure/evening time (question 2) was calculated from published values for energy costs for walking and cycling [31].Black et al derived the PAL values for different categories of work from 500 measurements based on the doubly labeled water method and indirect calorimetry [30]. The published energy costs (so called metabolic energy transfer, or MET, values) are total energy expenditure when walking and cycling divided by basal metabolic rate. These values were derived from measurements of energy expenditure using indirect calorimetry in laboratory settings [31]. PAL_cell _was calculated as the mean of the 14 PAL values for days 1 through 14.

### Calculation of PAL From the Two Paper Questionnaires

Questionnaires 1 and 2 are displayed in Multimedia Appendix 1. Questionnaire 1 consisted of one simple question that has been used in previous epidemiological studies [38,39]. Each woman graded her physical activity level during the last two weeks between 1 and 10, where 1 is very low, and 10 is very high. Women were informed that the value 1 should be interpreted as a sedentary lifestyle, while the value 5 represented a few long walks per week, and the value 10 represented exercise several times a week. The answer was converted to PAL_quest1 _. Value 1 represented a PAL value of 1.3. Each step up to 10 represented a 0.1 increase up to 2.2.

Questionnaire 2 required each woman to report the number of hours and minutes spent in nine different activity categories with assigned MET values [38] during one average 24-hour period during the last two weeks [8]. PAL_quest2_ was calculated as MET times the number of minutes for each activity category divided by 1440 minutes.

### Calculation of AEE

For each woman, PAL_cell_, PAL_quest1_, and PAL_quest2_ were converted to corresponding measures of activity energy expenditure (AEE), that is AEE_cell_, AEE_quest1_, and AEE_quest2_, and were calculated as (PAL times basal metabolic rate) minus basal metabolic rate. Reference estimates of AEE (AEE_ref_) were calculated as total energy expenditure minus basal metabolic rate.

### Statistical Analysis

Values are given as mean ± SD. Significant differences between mean values were identified by repeated measures analysis of variance (ANOVA) with subsequent post hoc analyses using Tukey’s multiple comparison tests after having ascertained that the values were normally distributed. The agreement between PAL_cell_, PAL_quest1 _, and PAL_quest2 _versus PAL_ref _was evaluated using the Bland and Altman procedure [40]. This procedure is used to evaluate the agreement between estimates obtained using an alternative method and estimates obtained using a reference method. According to the procedure, the difference between the estimates obtained from the alternative and reference methods (y) was plotted against the average of these two estimates (x) for all subjects. The mean difference ± 2SD for all subjects was then calculated. Two SD was chosen as the measure of variation since this estimate corresponds to the limits of agreement used by Bland and Altman. To test for trend within methods, a linear regression model was fitted between x and y. The same procedure was used for AEE values. Intraclass correlation was used to measure intraindividual variation of daily physical activity levels through cell phones. All statistical tests were done on a two-sided 5% level of significance. All statistical analyses were performed using SAS 9.1.3 (SAS Institute Inc, Cary, NC, USA).

## Results

### Characteristics of Participants

Characteristics of women who participated in the study are shown in Table 2. The women represented a wide range of age, body mass index (BMI), and body weight. Of the 22 participating women, 4 had a BMI greater than 25 kg/m^2^, and 2 had a BMI greater than 30 kg/m^2^. None of the women were smokers, and all but 4 had a university or college degree. At baseline, 5 women (23%) reported that they never exercised, 5 women (23%) reported that they exercised 1 to 2 times per week, while 12 women (54%) reported that they exercised 3 times a week or more.

**Table 2:** Characteristics of the 22 women in the study

Characteristic	Mean ± SD	Range	Median	Q1^a ^	Q3^b ^
Age (years)	35.1 ± 8.3	20-45	37	29	42
BMI (kg/m^2 ^)	23.7 ± 3.8	17.7-33.6	22.1	20.5	25.1
Height (m)	1.69 ± 0.06	1.55-1.81	1.69	1.65	1.74
Bodyweight (kg)	67.2 ± 13.3	47-102	65.5	58.7	73.4
Exercise (hours/week during the last year)^c ^	2.4 ± 1.8	0-5	3	1	4

^a^First quartile: cutoff for lowest 25%

^b^Third quartile: cutoff for lowest 75%

^c^Defined as regular exercise (eg, sports, aerobics or running) during the year before the study. Walking or cycling for transportation (eg, to or from work) was not included.

### Procedure

Of the 22 women, 14 (64%) had a cell phone that supported the Java-based application, and 8 (36%) borrowed a cell phone from the study center. During the 14-day period, all women but 2 answered all questions. Of the 2 women who did not answer all questions, one failed to report once, and the other, twice. For these women, PAL_cell_was based on 13 and 12 days of reporting, respectively. None of the women asked to terminate the reporting before the end of the 14-day period, and at the end of the study, all women expressed little or no burden of completing the reporting during the 14-day period.

### PAL Assessed Using Cell Phones

**Figure 2:**
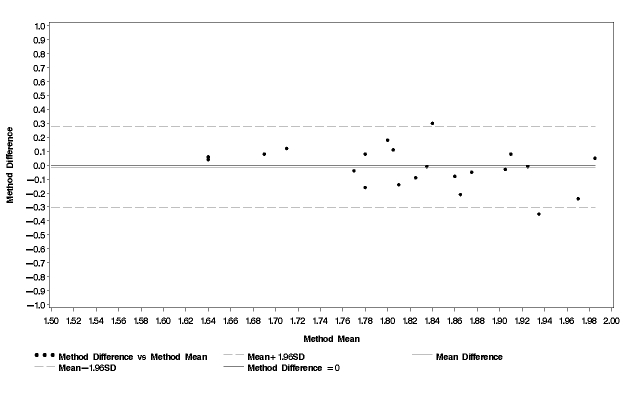
Bland and Altman plot comparison of physical activity level obtained using cell phones during 14 days (PAL_cell_) and physical activity level obtained using a combination of the doubly labeled water method and indirect calorimetry (PAL_ref_)

Table 3 demonstrates total energy expenditure and basal metabolic rate as well as PAL obtained using different methods. On average, PAL_cell _was 1.82. This value was not statistically significantly different from PAL_ref _, which was 1.83. The Bland and Altman plot for PAL_cell _in comparison with PAL_ref _is shown in Figure 2. The mean difference for PAL_cell _and PAL_ref _was small (0.014), and the limits of agreement were 2SD = 0.29. The test for trend was not statistically significant. The regression equation was y = -0.58 x + 1.05;*r *= -0.38;*P *= .08.

**Table 3:** Total energy expenditure,basal metabolic rate, and physical activity level (PAL) obtained using different methods in 22 Swedish women

Measurement	Mean ± SD	Range	Median	Q1^a ^	Q3^b ^
Total energy expenditure(kJ/24h)	10810 ± 1410	8130-13120	10640	9960	11620
Basal metabolic rate(kJ/24h)	5900 ± 710	4920-7950	5840	5490	6250
PAL_ref _	1.83 ± 0.14	1.61-2.11	1.86	1.71	1.92
PAL_cell _	1.82 ± 0.10	1.66-2.01	1.82	1.75	1.89
PAL_quest1 _	1.84 ± 0.23	1.50-2.20	1.85	1.60	2.00
PAL_quest2 _	1.90 ± 0.43	1.47-3.01	1.75	1.54	2.18

^a^First quartile: cutoff for lowest 25%

^b^Third quartile: cutoff for lowest 75%

### PAL Assessed Using Paper Questionnaires

**Figure 3:**
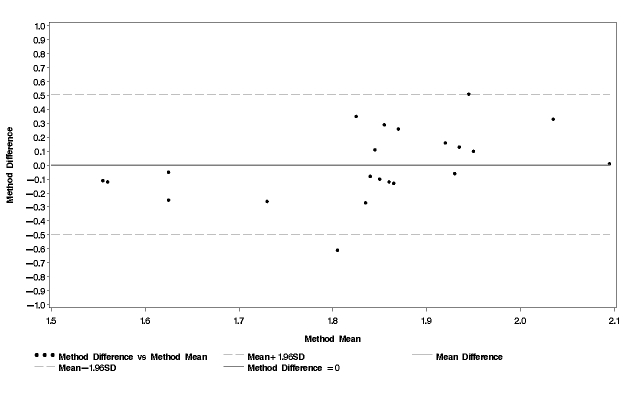
Bland and Altman plot comparison of physical activity level obtained using the first questionnaire (PAL_quest1_) and physical activity level obtained using a combination of the doubly labeled water method and indirect calorimetry (PAL_ref_)

**Figure 4:**
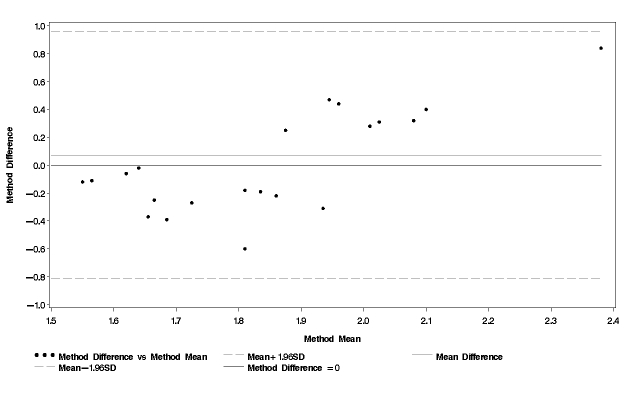
Bland and Altman plot comparison of physical activity level obtained using the second questionnaire (PAL_quest2_) and physical activity level obtained using a combination of the doubly labeled water method and indirect calorimetry (PAL_ref_)

The average PAL_quest1_was 1.84 while the average PAL_quest2_was 1.90 (Table 3). None of these values was statistically significantly different from PAL_ref_. The Bland and Altman plots for PAL_quest1_and PAL_quest2_in comparison with PAL_ref_are shown in Figure 3 and Figure 4. The mean difference was small for both PAL_quest1_and PAL_quest2_compared with PAL_ref_(0.004 and 0.07 respectively). However, the limits of agreement were wider than for PAL_cell _(PAL_quest1 _, 2SD = 0.51 and PAL_quest2 _, 2SD = 0.90). The test for trend was statistically significant for PAL_quest1_(the regression equation was y = 0.79 x - 1.45; r = 0.44; *P*= .04) as well as for PAL_quest2_(the regression equation was y 1.58 x - 2.88; *r*= 0.65; *P*< .001). Thus, both questionnaires (in particular questionnaire 2) overestimated higher PAL values while they underestimated lower PAL values.

### AEE From Cell Phones and Paper Questionnaires

AEE_cell_, AEE_quest1_, and AEE_quest2_versus AEE_ref_ are shown in Table 4. The results were very similar to results for the PAL estimates. Similar results were obtained when the results were expressed as AEE in kJ/24h and per kg body weight (data not shown).

**Table 4:** Comparison of activity energy expenditure (AEE) assessed by cell phones and questionnaires in relation to reference estimates in 22 Swedish women

Measurement	Mean Difference (kJ/24h)	2SD for the Mean Difference (kJ/24h)	*r*^e^	*P*^f^
AEE_cell _^a^- AEE_ref_^b^	95	2380	-0.11	0.64
AEE_quest1 _^c^- AEE_ref _	200	3630	0.42	0.05
AEE_quest2 _^d ^-AEE_ref _	540	4980	0.73	< .001

^a^AEE_cell_= Activity energy expenditure obtained using the cell phone

^b^AEE_ref_= Total energy expenditure minus basal metabolic rate

^c^AEE_quest1_= Activity energy expenditure obtained using questionnaire 1

^d^AEE_quest2_= Activity energy expenditure obtained using questionnaire 2

^e^The correlation coefficient for the linear regression between the average of the alternative method and the reference method and the difference between them. For instance, the *r* value when AEE_cell_- AEE_ref_ was regressed on the average of AEE_cell_ and AEE_ref_.

^f^ *P*-value for the *r*-value

### Day-to-Day Variation in PAL Obtained Using Cell Phones

**Figure 5:**
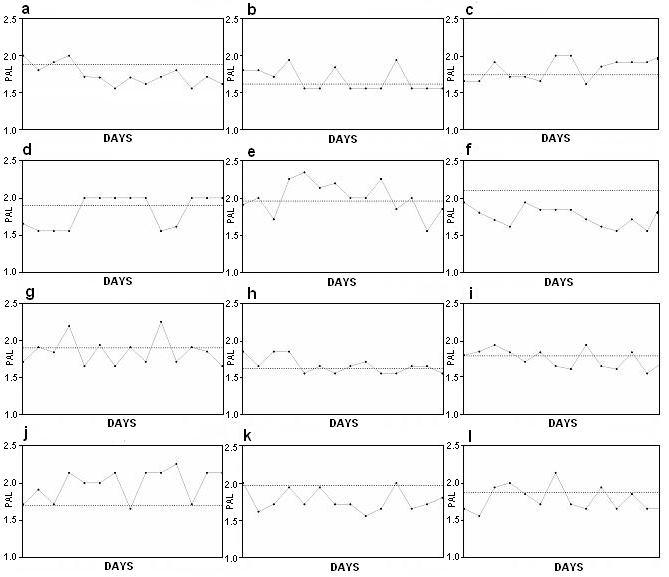
Daily PAL values obtained using cell phones during day 1 through day 14 for twelve selected women compared with PAL_ref_ (covering the whole 14-day period and shown as a straight dotted line for each woman)

PAL obtained using cell phones varied considerably from day to day during the 14-day study period (Figure 5). The intraclass correlation coefficient for the 22 women was estimated to be 0.20; thus about 20% of the variation is between women, while about 80% of the variation is due to day-to-day differences.

## Discussion

This study describes a novel approach to collecting data on physical activity using a Java-based physical activity questionnaire administered repeatedly through cell phones. The results indicate that measuring physical activity through cell phones is a promising method of assessing PAL that could be used in large-scale epidemiological studies.

The method generated high compliance and high acceptance among the participants. On average, PAL obtained using cell phones agreed well with reference estimates of PAL obtained using the doubly labeled water method and indirect calorimetry. Also the PAL values assessed by means of the two paper questionnaires were in good agreement with reference estimates. However, the limits of agreement for the difference between PAL obtained by cell phone and reference PAL were narrow (2SD = 0.29), while the corresponding limits for the two paper questionnaires were much wider (2SD = 0.51 for questionnaire 1 and 2SD = 0.90 for questionnaire 2). Furthermore, both questionnaires produced biased results (especially questionnaire 2), overestimating PAL of physically active women while underestimating PAL of less active women.

Only two previous studies have compared PAL estimates obtained from paper questionnaires to reference estimates for healthy adults based on the doubly labeled water method and indirect calorimetry. In these studies, paper questionnaires underestimated PAL by 0.12 units (6%) [33] or overestimated PAL by 0.31 units (31%) [41]. In our study, the cell phone estimates agreed by 0.01 units or 1% compared with the reference estimates on average. Only one of the former studies reported 2SD of the difference between PAL obtained using paper questionnaires and reference estimates. Their limits of agreement were wider than for the cell phone estimates in this study (2SD being 0.64 compared with 0.29) [33].

When expressing the results as AEE, the cell phone questions overestimated reference estimates of AEE by only 2% on average. For comparison, in the recent review by Neilson et al [15], only eight of twenty studies reported a mean difference in total energy expenditure or AEE less than 10%, and only four reported a mean difference less than 2% compared with reference estimates. Two SD was 2380 kJ/24h for the difference between AEE_cell_ compared with AEE_ref_. These limits of agreement are narrower than for most paper questionnaires that have been evaluated previously using reference estimates [15].

Our results showed a minor tendency (although not statistically significant) for PAL to be underestimated for physically active women using the cell phone questionnaire (Figure 2). This may be explained by the fact that in the current version, leisure activities included only walking, cycling, and sports. Other common everyday activities like gardening, moving, or playing with children may also increase PAL. Such activities could be included in a future version to test if they improve the ability of the cell phone questions to assess PAL of individual women. But the underestimation might also be explained by a skewed scale in the cell phone questionnaire, and that the options that require a higher level of physical activity should be given higher values.

Many paper questionnaires are designed to assess AEE. When comparing AEE between individuals, it should be expressed in kilojoules per kg body weight since smaller individuals have smaller AEE. This is a concern in epidemiological studies where body weight is often self-reported, creating a risk of bias. PAL is independent of body weight. In this study, PAL was chosen since our overall goal was to develop a procedure for use in epidemiological settings. Reference estimates of PAL were assessed as total energy expenditure divided by basal metabolic rate. Total energy expenditure consists of basal metabolic rate (50-70%), energy expenditure due to physical activity (20-40%), and diet-induced thermogenesis (5-10%). Thus, in the calculations of PAL, diet-induced thermogenesis was included. However, the cell phone and questionnaire estimates of PAL were calculated using published values for PAL [30] and MET [31], which also included diet-induced thermogenesis.

Figures 3 and 4  indicate that both questionnaires overestimated PAL of active women and underestimated PAL of more sedentary women. These results were obtained using the average of the alternative and reference method on the x-axis as described by Bland and Altman [40]. However, in some studies only the standard method is used on the x-axis. Had this procedure been used, the correlations would be somewhat different. According to Bland and Altman, using only the reference value will likely show a correlation whether there is a true association between difference and magnitude or not [42]. Thus, in this study, the average value was used on the x-axis.

Only 2 of the 22 women missed a reporting occasion (one failed to report once, the other, twice), indicating a high compliance for the study method. The high compliance may be due to the SMS sent as a reminder. The technique also provides a possibility for the study center to monitor whether the participant answers the cell phone questions during the study period. These features are an advantage compared with ordinary paper-based physical activity records. None of the 22 women expressed any concerns or that having to report daily had interfered with daily life.

The two questions were downloaded onto cell phones using a Java-based application; thus the participants had to have access to a cell phone with Java support. Out of the 22 women, 14 (64%) were able to download the Java-based questionnaire onto their own cell phone while 8 (36%) borrowed a cell phone from the study center. The same distribution of access to cell phones supporting a Java-based questionnaire was seen in another unpublished study. The advantage with Java in Web-based applications is that more advanced applications can be used, and the cost of data transfer is lower than the cost of sending an SMS. Furthermore, the Java program can also store data if the online connection is poor and then send the data once the signal is stronger. Once the program had been downloaded on the participant’s cell phones, the time and effort from the study center was minimal as all communications were automatically managed by the application server. As the cell phone questionnaire included only two questions, the questions could be asked through SMS or interactive voice response (IVR) in order to increase the accessibility, though these techniques allow less advanced interface and are more dependent on connection to the cell network. However, both these techniques are accessible to all cell phones and have been used to communicate with patients in intervention and therapy studies [17,21,43].

The strengths of this study are that this is the first study to have developed and evaluated a cell phone based method of assessing PAL, and that gold standard methods were used as reference methods. One possible limitation of the PAL cell phone estimates is that they were based on self-report. Still, the mean difference ± 2SD between PAL obtained using cell phones and reference estimates was smaller than earlier studies that reported evaluations of PAL or total energy expenditure that had been assessed using objective methods like accelerometry [33,44]. The present study is small, with only 22 participants, but the study participants represented a wide range of BMI, exercise habits, and age. They were also a group of well-educated, highly motivated, and moderately active women. The results might not be applicable to other populations or subgroups in other social settings with a different age distribution or different levels of physical activity.

In this study high within-subject variation in relation to between-subject variation was noted for the cell phone estimates. The high within-subject variability is a natural consequence (although not shown earlier in this way) of subjects having different levels of physical activity from day to day, but it may also indicate that the cell phone questionnaire may not have captured all of the information about subjects’ physical activity. We aimed to develop a simple procedure to classify physical activity levels of subjects suitable for epidemiological studies that could predict health outcomes many years later. For this purpose, short-term (day-to-day or hour-to-hour) variation is likely not a problem. However, our procedure may need modification to be useful for intervention studies since such studies may require more detailed information, such as the type and duration of physical activities performed. We cannot exclude the possibility that the low between-subject variation was to some extent due to the fact that our study participants were a relatively small group of women. However, this study is a first step toward developing and evaluating a unique cell phone strategy to assess PAL. These first results in a group of healthy Swedish women are promising, and the next phase should include evaluations in other populations including men and different age groups.

Using the procedure described by Bland and Altman, the cell phone estimates produced narrow limits of agreement when compared with reference estimates. We can only speculate why, but it may be due to the fact that the cell phone questions were answered every evening, reducing the reporting error due to loss of memory. Another reason may be that the procedure takes advantage of a technique (cell phones) that is associated with “instant answers” which also may reduce reporting error. Paper questionnaires may provide subjects with more time to reflect on their answers and also to consider their total activity level, which may make subjects more prone to adjust their answers in order not to be “too inactive,” for example.

Noteworthy is that the more detailed paper questionnaire used in this study was not superior to the use of only one rating question in assessing total physical activity. This finding supports the result from a recent study in rheumatoid arthritis patients, where good agreement was found for PAL values obtained through two questions on physical activity compared with reference estimates [36]. Future studies should evaluate if these results are also valid for other populations including men and other age groups.

In this study, large day-to-day variations in PAL were shown, indicating the need for repeated measurements within subjects to decrease this variation. When repeating the questions, it was possible to obtain average PAL estimates typical for individuals. Furthermore, this study shows that cell phones are useful tools for repeated collection of PAL values. This procedure for gathering information about physical activity has a great potential for large-scale prospective epidemiological studies. The cell phone-based procedure could also be used for data collection of other variables in epidemiological settings where variation could be decreased by repeated measurement, such as assessment of energy intake or other health-related variables.

In conclusion, a Java-based physical activity questionnaire administered using cell phones produced average PAL estimates that agreed well with PAL reference values. Furthermore, the limits of agreement between PAL obtained using cell phones and reference values were narrower than for the corresponding estimates obtained using paper questionnaires. Java-based questionnaires downloaded onto cell phones may be a feasible and cost-effective method of data collection for large-scale prospective studies of physical activity.

## Multimedia Appendix

The two paper questionnaires used in the study

## Abbreviations

AEE: activity energy expenditure
BMI: body mass index
https: hypertext transfer protocol secure
IVR: interactive voice response
Java: Java technology was created as a computer programming tool at Sun Microsystems in 1991
MET: metabolic energy turnover
PAL: physical activity level
SD: standard deviation
SMS: short message service
