# Supplementary material for: Measures of Physical Activity Using Cell Phones: Validation Using Criterion Methods
Source: J Med Internet Res. 2010 Jan 29;12(1):e2. doi: 10.2196/jmir.1298 (PMC2821583; doi:10.2196/jmir.1298)
Supplement: Supplementary file 1 [file jmir_v12i1e2_app1.pdf]

Multimedia Appendix 1. The two paper questionnaires used in the study

1 - Very low ☐ ☐ ☐ ☐ ☐ ☐ ☐ ☐ ☐ ☐ **10 - Very high**

Grade your physical activity during the last two weeks. The value 1 should be interpreted as a sedentary lifestyle, while the value 5 represents a few long walks per week, and the value 10 represents exercise several times a week.

2. Fill in the number of hours and minutes that you spent on average in each activity category A to I per day during the last two weeks.

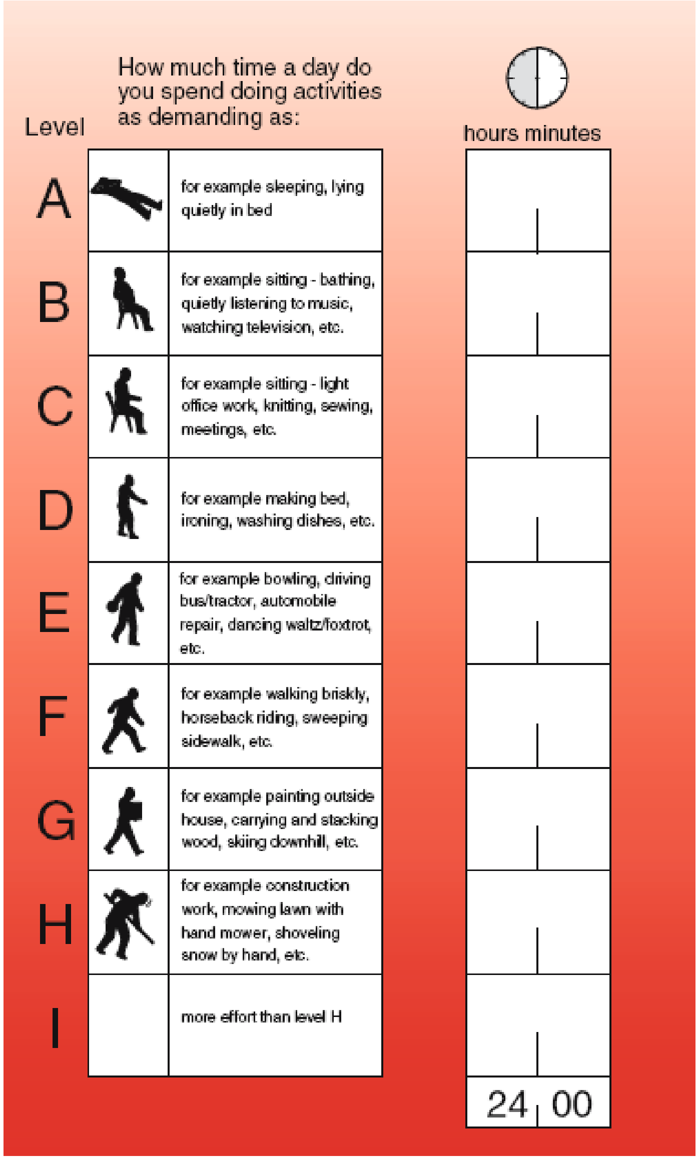

Level	How much time a day do you spend doing activities as demanding as:	hours minutes
A	for example sleeping, lying quietly in bed	
B	for example sitting - bathing, quietly listening to music, watching television, etc.	
C	for example sitting - light office work, knitting, sewing, meetings, etc.	
D	for example making bed, ironing, washing dishes, etc.	
E	for example bowling, driving bus/tractor, automobile repair, dancing waltz/foxtrot, etc.	
F	for example walking briskly, horseback riding, sweeping sidewalk, etc.	
G	for example painting outside house, carrying and stacking wood, skiing downhill, etc.	
H	for example construction work, mowing lawn with hand mower, shoveling snow by hand, etc.	
I	more effort than level H	
		24 00
